# Parasite-host relationships of water mites (Acari: Hydrachnidia) and black flies (Diptera: Simuliidae) in southeastern Spain

**DOI:** 10.1186/s13071-022-05610-2

**Published:** 2022-12-17

**Authors:** David López-Peña, Reinhard Gerecke, Eduardo Moisés García-Roger, Peter Martin, Ricardo Jiménez-Peydró

**Affiliations:** 1 Entomology and Pest Control Laboratory, Institut Cavanilles de Biodiversitat I Biologia Evolutiva (ICBiBE), Universitat de València (Estudi General), C/ Catedrático José Beltrán Martínez, 2, 46980 Paterna, Valencia Spain; 2 Department of Evolution and Ecology, Faculty of Science, University of Tübingen, 72076 Tübingen, Germany; 3 Evolutionary Ecology Laboratory, Institut Cavanilles de Biodiversitat I Biologia Evolutiva (ICBiBE), Universitat de València (Estudi General), C/ Catedrático José Beltrán Martínez, 2, 46980 Paterna, Valencia Spain; 4 Department of Landscape Ecology, Institute for Natural Resource Conservation, Christian-Albrechts-Universität Zu Kiel, Olshausenstr. 75, 24118 Kiel, Germany

**Keywords:** Black flies, Water mites, Physico-chemical variables, Abundance of pupae, Prevalence and intensity of parasitism, Parasitic load, Eastern Spain

## Abstract

**Background:**

Documentation on water mites in Spain is scarce, as is information on the parasite-host relationship between certain water mite species and representatives of the dipteran family Simuliidae. The discomfort caused to humans and animals by black flies seems to be increasing in recent years. In this context, an investigation of parasitic water mites is of great importance, not only from the point of view of biodiversity, but also in terms of their potential to control black fly populations.

**Methods:**

Rivers across a wide region of eastern Spain were sampled to determine the specific richness of simuliid dipterans and to investigate their possible parasites, such as water mites, mermithid nematodes and microsporidia (fungal microbes). Data on environmental variables, abundance, prevalence and intensity of parasitism on the collected specimens were analyzed.

**Results:**

In 10 streams, 15,396 simuliid pupae were collected and checked for the presence of water mite larvae; 426 pupae in seven streams were found to be associated with water mite larvae. Of the 21 simuliid species identified based on morphological characters, eight were found to be associated with water mite larvae. Water mite infection was not equally distributed among black fly species. Also, the prevalence of parasitism was low and differed among simuliid species, ranging from one to 13 water mites per black fly pupa. Variation at the intra- and interspecific levels was detected in terms of the number of water mites inside the black fly cocoons. Free-living deutonymphal and adult water mites representing 15 different species of six genera and five families were morphologically identified. The taxonomic identity of the parasitic mite larvae is unclear at present. Morphologically, they fit descriptions of larval *Sperchon* (*Hispidosperchon*) *algeriensis* Lundblad, 1942, but the possibility cannot be excluded that they represent* Sperchon algeriensis*, the most abundant species at the adult stage in this study and unknown at the larval stage, or even another species of the genus. A molecular analysis produced for the first time cytochrome oxidase I gene sequences for *S. algeriensis*.

**Conclusions:**

Our results contribute to current knowledge on Spanish Hydrachnidia and their relationships with simuliids as hosts. However, further research is needed to evaluate the diversity, distribution, bioecology and prevalence of this parasitism.

**Graphical Abstract:**

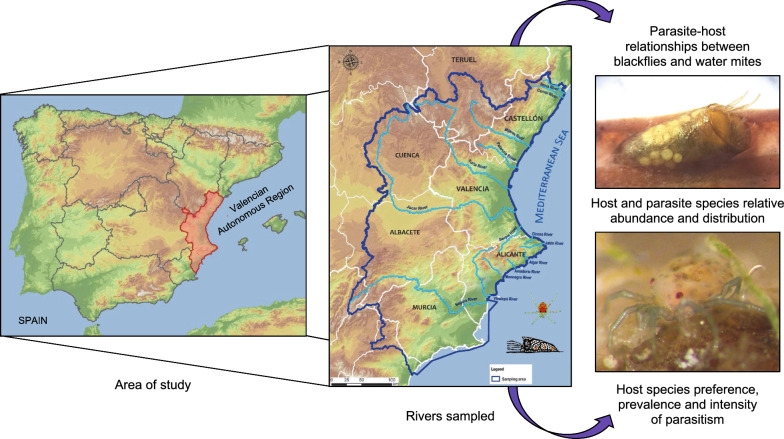

## Background

Mites are a taxonomic group that has achieved great success at adapting to almost any kind of habitat. High numbers of species are reported both from terrestrial and aquatic environments, from freshwater to seawater. Mites are able to distribute in many different ways, with some species even having developed structures that allow them to glide through the air [1]. Adaptation has also resulted in a wide radiation of feeding types, ranging from the ancestral predatory mode to phytophagy, saprophagy, mycophagy, necrophagy or parasitism [1, 2].

Parasitic water mites may play an important role in the natural control of insect populations [1, 3]. In this context, these arthropods may function as biological controllers of harmful organisms [1], as is the case of black flies, and could be considered as a potential tool in the control of various insect populations [4, 5]. Currently, there is an increasing interest in this type of biological control [6, 7]. However, it is important to take into account that the immense majority of water mites are found only in streams with year-round flow; consequently, simuliids are safe from water mites in intermittent streams, where many species are adapted to a seasonal water shortage. The eggs of some species can remain viable in a state of diapause for up to 2 years after oviposition in the dry beds of temporary water courses, which are typical of markedly seasonal climates in semi-arid zones. A precondition for the long-term survival of these eggs is shelter in the deepest and most humid strata of the interstices of the sediment [8, 9].

The life-cycle of water mites consists of seven stages: egg, prelarva, larva, protonymph, deutonymph, tritonymph and adult. Of these, only the larval, deutonymph and adult stages are active [10–12]; that is, they swim and crawl to locate appropriate hosts or prey—larvae as parasites, deutonymphs and adults as predators. Among the preferred hosts of larval water mites are the imaginal stages of the diptera [12–15], of which a special case are the black fly larvae parasitized by some species of the water mite genus *Sperchon* (most published data refer to *Sperchon* (*Hispidosperchon*) *setiger* Thor, 1898). While the larvae of most *Sperchon* species are known to use exclusively, or additionally, chironomids as their hosts [16–18], most records refer to *S. setiger* larvae parasitizing adult black flies [13]. The typical life-cycle of water mites has larvae present inside the cocoon harboring the black fly pupa during metamorphosis [19, 20]. These larvae wait until emergence of the black fly imago and then anchor to it, emerging into the open air with their host. After the adult black fly emerges into the air, the mite larvae feed by sucking the host’s body liquids. As parasites, water mites have an additional benefit in attaching to hosts since this attachment is also a means to increase dispersion [13]. When adult black flies approach the water, for example, to lay eggs, mite larvae return to the water [12, 21, 22] and continue progressing through their own life-cycle over several molts to the adult stage.

As many *Sperchon* larvae have been found to be remarkably engorged during their stay in the cocoon, Renz et al*.* [20] suggest that they are also able to feed before the host emerges. In this case, *Sperchon* larvae parasitizing simuliid pupae are a rare exception in the typical mode of water mite parasitism that predominantly targets insect adults. It is currently unclear how this food uptake takes place (lesions in the hosts integument are found only in exceptional cases). Should *Sperchon* larvae be able to complete their life-cycle without parasitism on adult hosts, then they would leave the black fly cocoon for further development—in no case would protonymphal *Sperchon* then be found inside cocoon. In the same study, Renz et al. [20] also tried to attract *Sperchon* larvae to simuliid larvae, but the latter were ignored completely by mites. Deutonymphal and adult water mites of most species feed by sucking the body fluids of small invertebrates or their eggs following their injection of digestive enzymes [23–25]. A special prey preference for black fly larvae was reported by Ullrich [26] and Martin [25] for water mites of the species *S. setiger*.

There are still many gaps in our knowledge of the relationship between water mites and black flies. A main limitation is that most research on black fly parasitism by water mite larvae has been attributed to one species of water mite only, namely *Sperchon setiger*. However, the larval morphology and hosts of many other species of the genus remain as yet unknown, and it cannot be excluded that a wider range of sperchontids have developed a host preference for simuliids. In this context, recent molecular studies (Stur and Gerecke, unpublished) suggest that other related *Sperchon* species coexisting with *S. setiger* might be simuliid parasites as well, and that even *S. setiger* in the classic sense might represent a mix of two or more cryptic species.

In the present study, simuliid-parasitic water mite larvae were found in habitats from which *S. setiger* was collected in low numbers, in association with strong populations of *Sperchon* (*Hispidosperchon*) *algeriensis* Lundblad, 1942, a related species as yet relatively unknown regarding its larval morphology and host preference. Unfortunately, our attempt to use molecular methods to determine if the simuliid-parasitic mite larvae might belong to the latter species was unsuccessful. However, with the support of Vladimir Pešić (Podgorica), in the present study we were able to obtain for the first time *S. algeriensis* cytochrome oxidase I gene (COI) sequence data. The results of the present study may contribute towards elucidating this complex question in the near future. At the present time, we can state that the identification of the mite larvae treated in our study seems to agree with that by Ullrich [27] for *S. setiger* larvae; however, given the general uncertainty of the taxonomic situation, we use “*Sperchon* sp.” in the study. Nearly all *Sperchon* species known worldwide are reported from running waters, the habitat to which simuliid larvae are perfectly adapted.

In the study reported here, we specifically address the following questions: (i) How prevalent is water mite parasitism on pupae of black flies in the field? (ii) Do water mites display any preference for parasitizing certain black fly species? (iii) Does the prevalence (i.e. fraction of parasitized hosts) and intensity (i.e. the mite load in hosts) of water mite parasitism vary geographically or in association to local ecological conditions of streams and rivers? Finally (iv), in order to better know the potential sources of black fly parasites: what is the composition of water mite assemblages in the field?

## Methods

### Study area and sampling design

**Fig. 1:**
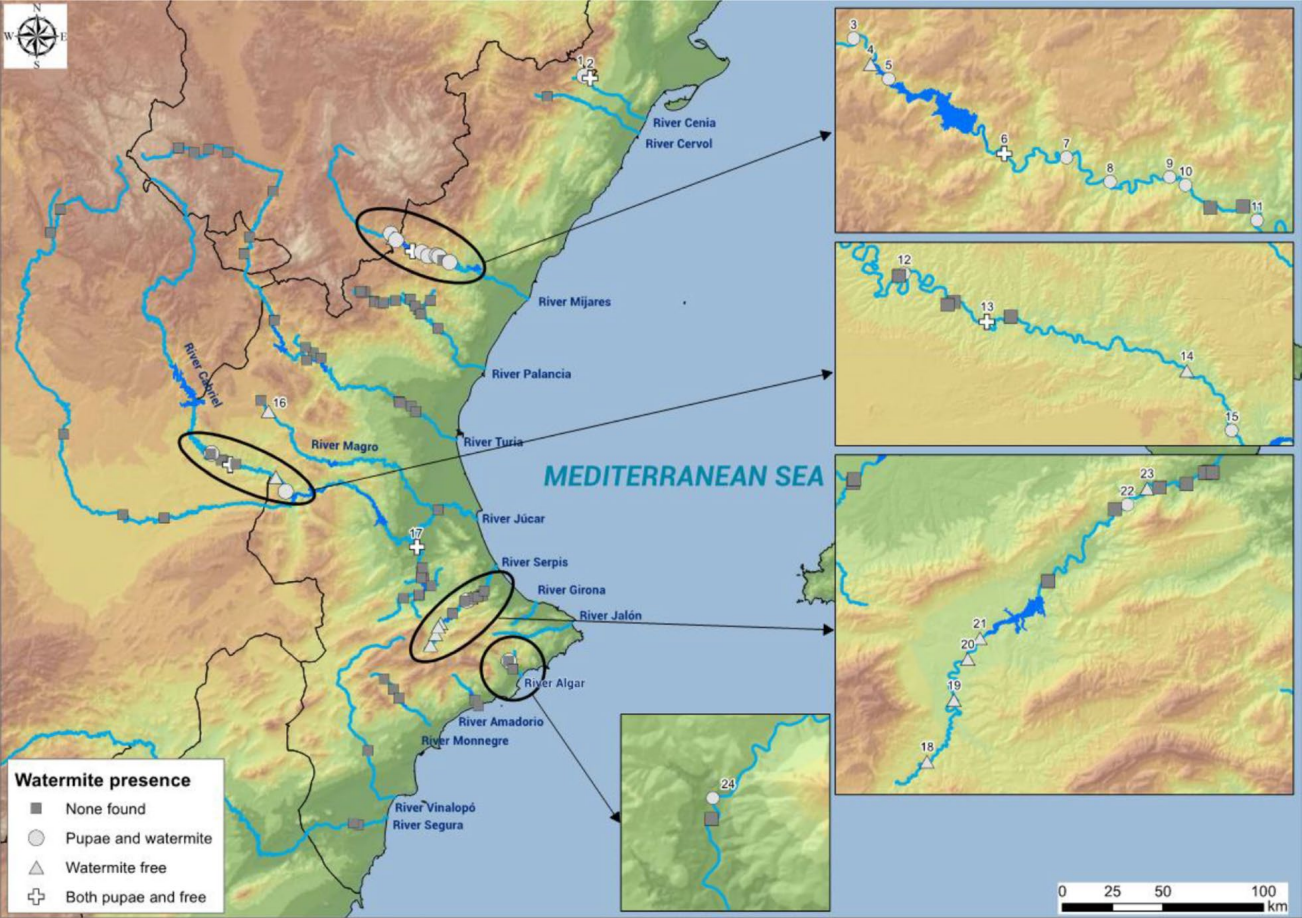
Sampling points where water mites were collected

In order to address the issues outlined above, a study area was chosen in eastern Spain, comprising mainly the three provinces that form the Autonomous Region of Valencia, but also the adjacent provinces of Albacete and Cuenca (Castilla-La Mancha) and Teruel (Aragón) (see Fig. 1). It should be noted that the prevalence and intensity of water mite parasitism on black flies will reflect the conditions in the study area, but we expect that, with due caution, the patterns that will emerge could be extended to other geographic areas and inspire future work to support our findings. The study area has already been the subject of an intensive investigation on the abundance and ecological preferences of black fly species, especially those of most concern at the biomedical and veterinary levels [28]. Based on data from this earlier study, we investigated 14 rivers and their tributaries in the present study. During the study period, two of these 14 rivers were completely dry (Girona and Jalón rivers) and the Segura and Vinalopó rivers were negative for the presence of both simuliids and water mites. Sampling sites ranged in latitude from the Senia river in the north (N 40º40′17.6″; E 0º14′20.1″) to the Algar river in the south (N 38º39′35,5"; W 0º5′52,5"), and in elevation from 88 to 664 m a.s.l. Samplings took place between 27 June 2013 and 7 August 2015.

At each sampling site, the following physico-chemical variables of the water were measured in situ: temperature (ºC), pH, dissolved oxygen concentration (mg//l) and its percentage of saturation (%), conductivity (μS/cm), turbidity (nephelometric turbidity units [NTU]), salinity (g/l), total dissolved solutes (TDS; mg/l) and redox potential (mV). Other local environmental variables, such as elevation, air temperature, mean particle size of streambed or the riparian vegetation, were also recorded since such factors have been shown to be useful in predicting aquatic insect distribution in streams [29, 30]. All of these collected data were used to construct an environmental variables’ dataset (Table 1), to be used in subsequent data analyses.

**Table 1:** Data of the physico-chemical variables in the samples where water mites were found

Sample number order	Date (day/month/year)	Locality	Province	River	Coordinates	Elevation (m a.s.l.)	Relative humidity (%)	pH	Redox potential (mV)	Electric conductivity (mS/cm)	TDS (g/l)	Salinity (g/l)	Turbidity (NTU)	Dissolved oxygen (%)	Water temperature(ºC)
1	09/07/2015	Pobla de Benifassà	Castellón	Cenia	N 40º40′17.6″ E 0º14′20.1″	417	44.6	8.35	− 67	4.33	2.37	2.2	0.39	55.1	22.7
2	10/07/2015	Rosell	Castellón	Cenia	N 40º39′46.8″ E 0º15′54.8″	381	47.6	8.75	− 91	5.5	3	2.7	0.4	66.3	20.5
3	10/07/2015	Rosell	Castellón	Cenia	N 40º39′46.8″ E 0º15′54.8″	381	47.6	8.75	− 91	5.5	3	2.7	0.4	66.3	20.5
4	22/07/2013	Olba	Teruel	Mijares	N 40º08′2.8" W 0º37′25.3"	664	56.6	7.78	− 37	0.50	0.28	0.2	7.23	72.8	18.2
5	20/01/2015	La Monzona	Castellón	Mijares	N 40º7′15.19″ W 0º36′46.7″	604	51.8	8.57	− 76	7.4	3.9	3.7	1.48	35.5	7.2
6	22/07/2013	Puebla del Arenoso	Castellón	Mijares	N 40º06′47.8″ W 0º36′5″	582	55	8.29	− 63	0.53	0.28	0.2	9.37	76	18.8
7	22/07/2013	Montanejos	Castellón	Mijares	N 40º04′26.1″ W 0º31′37.1″	458	31.8	7.92	− 46	0.75	0.4	0.4	2.34	65.8	28.5
7(2)	20/01/2015	Montanejos	Castellón	Mijares	N 40º04′0″ W 0º31′16.6″	448	38.4	8.46	− 73	8.1	4.4	4.1	1.27	42.9	17.9
7(3)	20/01/2015	Montanejos	Castellón	Mijares	N 40º04′0″ W 0º31′16.6″	448	38.4	8.46	− 73	8.1	4.4	4.1	1.27	42.9	17.9
8	22/07/2013	Arañuel	Castellón	Mijares	N 40º04′16.2″ W 0º29′10.4″	401	40.7	8.25	− 63	0.75	0.4	0.4	1.49	99.5	23.5
8(2)	27/01/2015	Arañuel	Castellón	Mijares	N 40º04′16.2″ W 0º29′10.4″	401	70.6	8.27	− 62	8	4.3	4	1.01	38.3	14
9	22/07/2013	Cirat	Castellón	Mijares	N 40º03′29.8″ W 0º27′29.5″	369	45.8	8.45	− 73	0.73	0.39	0.4	2.47	86.1	23.5
9(2)	27/01/2015	Cirat	Castellón	Mijares	N 40º03′29.8″ W 0º27′29.5″	369	59.4	8.33	− 64	8.4	4.5	4.2	1.27	38.7	10.9
10	29/07/2013	El Tormo	Castellón	Mijares	N 40º03′35.4″ W 0º25′8.3″	327	49.5	7.87	− 43	0.53	0.28	0.2	2.67	104.9	20.4
11	29/07/2013	Torrechiva	Castellón	Mijares	N 40º03′19.9" W 0º24′31.9″	313	49.5	7.85	− 42	0.54	0.29	0.2	2.28	83.3	20.1
12	29/07/2013	Toga	Castellón	Mijares	N 40º02′12.1" W 0º21′45.4"	263	37.3	7.86	− 42	0.55	0.29	0.3	2.66	96.7	21.8
13	21/07/2015	Villatoya	Albacete	Cabriel	N 39º22′44.5″ W 1º25′30.7″	426	41.4	8.47	− 73	6.7	3.5	3.3	2.7	52.1	18
14	21/07/2015	Villatoya	Albacete	Cabriel	N 39º20′22.6″ W 1º20′29.5″	364	29.7	8.42	− 71	6.8	3.7	3.5	2.09	52.2	20.3
15	05/05/2015	Aielo de Malferit	Valencia	Clariano	N 38º52′43.4″ W 00º34′3.8″	221	47.4	8.92	− 99	5.7	3	2.8	11.38	52.5	22.8
16	23/07/2015	Salto de Cofrentes	Valencia	Cabriel	N 39º14′58.1″ W 1º5′38.3″	327	50.9	8.62	− 81	6.8	3.6	3.4	12.34	50.8	20
17	20/05/2015	Genovés	Valencia	Albaida	N 38º59′6.2" W 00º29′6.0"	88	79.3	8.21	− 60	5.14	2.8	2.15	20.7	46.6	19.2
18	23/07/2015	Casas del Río	Valencia	Cabriel	N 39º17′51.4″ W 1º8′18.2″	353	64	8.45	− 73	6	3.2	2.7	9.62	50.8	19.1
19	25/07/2013	Villalonga	Valencia	Serpis	N 38º53′20.06″ W 00º13′8.3"	88	56.3	8.38	− 71	0.38	0.20	0.2	3.97	104.1	20.7
20	25/07/2013	Villalonga	Valencia	Serpis	N 38º53′18.1" W 00º13′18.9″	88	61	8.46	− 74	0.39	0.20	0.2	3.1	106.2	21.5
20(2)	27/04/2015	Villalonga	Valencia	Serpis	N 38º53′16.9″ W 00º13′19.4″	88	73.1	8.78	− 88	6.2	3.3	3	3.69	45.3	16.9
21	25/07/2013	Villalonga	Valencia	Serpis	N 38º53′18.5″ W 00º12′58.2″	88	58.1	8.58	− 81	0.39	0.21	0.2	3.48	110.5	22.6
21(2)	03/03/2014	Villalonga	Valencia	Serpis	N 38º53′18.5″ W 00º12′58.2″	88	–	8.73	− 85	0.31	0.17	0.1	4.51	-	14.7
21(3)	24/04/2014	Villalonga	Valencia	Serpis	N38º53′18.5″ W 00º12′58.2″	88	–	8.73	− 85	0.31	0.17	0.1	4.51	-	14.7
21(4)	27/04/2015	Villalonga	Valencia	Serpis	N 38º53′18.5″ W 00º12′58.2″	88	74.4	8.51	− 73	6.1	3.2	2.7	5.52	45.5	17.2
22	24/01/2014	Villalonga	Valencia	Serpis	N 38º52′13.3″ W 00º17′2.3″	168	58.1	6.26	41	0	0.02	0	2.67	-	11.4
22(2)	16/07/2015	Villalonga	Valencia	Serpis	N 38º52′13.3″ W 00º17′2.3″	161	56.9	8.92	− 99	6.2	3.3	3.2	14.67	52.5	24.8
23	16/07/2015	Villalonga	Valencia	Serpis	N 38º52′54.7″ W 00º14′12.6″	96	–	8.86	− 97	6.1	3.3	2.9	6.42	52.6	26.1
23(2)	27/04/2015	Villalonga	Valencia	Serpis	N 38º52′54.7″ W 00º14′12.6″	96	72.3	8.82	− 90	6.3	3.3	2.9	11.7	44.7	17
23(3)	30/06/2014	Villalonga	Valencia	Serpis	N 38º52′54.7″ W 00º14′12.6″	96	34.4	8.65	− 84	8.2	4.4	4.2	5.78	-	20.5
24	04/06/2014	L'Orxa	Alicante	Serpis	N 38º49′50.5″ W 00º21′5.7″	642	31.8	7.91	− 43	8.4	4.5	4.4	6.61	-	19.4
24(2)	03/03/2015	L'Orxa	Alicante	Serpis	N 38º49′50.5″ W 00º21′5.7″	642	48.6	8.72	− 87	8.2	4.4	4.1	98.3	40.5	11.9
25	29/06/2015	Callosa d'En Sarrià	Alicante	Algar	N 38º39′35.5″ W 0º5′52.5″	110	35	8.34	− 66	3.78	2.02	1.9	1.55	61.8	20.1

*NTU *Nephelometric turbidity units,* TDS* total dissolved solutes

Pupae of black flies and water mite larvae found in the pupal cocoons were sampled following a protocol based on the recommendations of previous researchers [31, 32]. At each sampling point, we chose a river section of 5–10 m in length where black fly pupae were collected from the substrate (i.e. cobbles, pebbles, tree branches, tree leaves, helophytes or submerged macrophytes) during a 15-min period while walking from one bank to the other. The sampling time invested in each single substrate type was proportional to its relative abundance in the river section. No net was used to specifically collect water mites, but the same sort of methodology was implemented to collect both the host and the parasite. The samples were brought to the laboratory where each sample (1 sample being the set of all specimens collected at a sampling point) was processed by first detaching the black fly pupae from the different substrates to which they were fixed. During the identification of pupae (for details, see López-Peña et al. [33]), water mite larvae were preserved in 80% ethanol, together with the black fly pupae that they were parasitizing, for later identification. Free-living adults and deutonymphs of water mites were also collected from all types of substrates in order to characterize the natural assemblages from which parasites could arise.

### Identification of water mite and black fly species

Water mite species identification was based on morphological descriptions in several taxonomic keys and the bibliography cited therein (for adults, see references [11, 34, 35]; for larvae, see references [26, 36–38]). Once identified, the specimens were stored in pure ethanol (collection Gerecke, Tübingen). In total, 922 mites were examined, among which were 80 free-living adults and deutonymphs; the remaining samples were larvae parasitizing the pupae of the black fly species collected. Selected water mite larvae (*n* = 5) and deutonymphs or adults (*Atractides* (*Atractides*) *nodipalpis* Thor, 1899 [*n* = 1]; *Aturus gallicus* Viets, 1939 [*n* = 2]; *Hygrobates* (*Hygrobates*) *fluviatilis* gr. (Ström, 1768) [ *n* = 1] *Lebertia* (*Pilolebertia*) *porosa* gr. Thor, 1900 [*n* = 1]; *Sperchon algeriensis* [*n* = 4]; *S.* (*Hispidosperchon*) *compactilis* Koenike, 1911 [*n* = 4]; *S. setiger* [*n* = 2], *Torrenticola* (*Torrenticola*) *barsica* (Szalay, 1933) [*n* = 1]) from the samples were sent for CO1 barcoding to the laboratory of Vladimir Pešić (University of Montenegro, Podgorica). Probably due to the sample age, only one of these specimens could be successfully sequenced, a male of *S. algeriensis*.

Identification of the black fly pupae at the species level was carried out using morphological taxonomic keys [39–41]. The samples are deposited in labeled vials in the Colección de Entomología de la Universitat de València (Estudi General). In total, 15,396 pupae were examined, of which 426 were parasitized by water mites.

### Data analysis

The prevalence of water mite parasitism on pupae of black fly species was investigated by describing, for each sample site and species, the fraction of the host population infected with parasites per sample site. The proportion of pupae of each black fly species infested with water mites was compared using generalized linear models (GLMs) with binomial responses that took into account the different rivers studied as grouping factor to search for eventual geographic patterns. The intensity of parasitism (i.e. mite load per host) was analyzed for parasitized black fly species using GLMs with Poisson distribution of errors and again considering the different rivers as a grouping variable. Because the GLMs were run in a species-by-species fashion, significance was corrected for multiple comparisons by applying Bonferroni’s method [42]. The correlation between prevalence and intensity of water mite parasitism was also explored, as well as their respective correlations with black fly abundance at each sampling point. The prevalence and intensity of water mite parasitism in each black fly species parasitized were also studied in relation to physico-chemical parameters describing local ecological conditions. Prior to analyses, the correlation among physico-chemical variables was explored to remove highly correlated variables (*R*^2^ > 0.8). The remaining variables were used as predictors in multiple logistic regression analyses for each parasitized black fly species, using binomial or Poisson responses for parasitism prevalence and intensity, respectively.

Finally, we assessed the similarity between species assemblages of free-living water mites according to the environmental variables of the sites where they were found. For this purpose, we followed a canonical ordination analysis approach [43]. As a first step, we used detrended canonical correspondence analysis (DCCA) to determine water mite species gradient lengths with respect to the same environmental variables as used in the principal component analysis (PCA) and, therefore, to assess whether unimodal (for further canonical correspondence analysis [CCA]) or linear-based (for further redundancy analysis [RDA]) models underlie the response of water mite species to environmental variables [44]. DCCA was performed on log-transformed data of water mite abundances at the adult and deutonymph stage per site and revealed a dominance of linear gradients (all maximal lengths < 3 standard deviations [SD] [45]), which enabled further analyses on taxonomic turnover in free-living water mites across ecological gradients to be based on RDA. Thus, RDA with forward selection was run to detect the main environmental variables that best explained the variability in water mite abundance. The significance of the variables introduced at each step was inferred from Monte Carlo permutation tests (999 permutations; *P*-value < 0.05), and model performance was assessed through the adjusted-R^2^ value.

All statistical analyses were carried out using the free software R version 3.3.3 from The R Foundation for Statistical Computing ([46] https://cran.r-project.org]. GLMs (including multiple logistic regression) and correlation analyses on the prevalence and intensity of water mite parasitism on black fly pupae were performed using the *glm* and *cor* functions, respectively, both available from the “stats” package [46]. DCCA and RDA on the assemblages of free-living water mites were performed using the *decorana*, *rda* and *ordistep* functions from package “vegan” [47].

### DNA extraction, amplification and sequencing

The barcode region of the COI of one male of *S. algeriensis* was sequenced using standard invertebrate DNA extraction [48], amplification [49] and sequencing protocols [50]. The COI data are deposited in the reference library of DNA barcodes of BOLD (The Barcode of Life Data System; https://www.boldsystems.org/), which provides a basis for building DNA barcode libraries at the regional and/or national level that contributes to the expansion of information on taxonomic, geographical and molecular species diversity, as well as on their distribution patterns. The voucher specimen is deposited in the collection Gerecke, Tübingen.

## Results

In total, 137 samples were collected from 94 sites distributed along 10 watercourses. Of these 137 samples, simuliids were found in 116 samples from 81 sites, and water mites were found in 37 samples from 25 sites. Likewise, water mite larvae were identified in pupal case pupae of Simuliidae in 18 samples from 12 sites of five rivers (Cenia, Mijares, Serpis and Algar rivers and Cabriel a tributary of Júcar river), free-living water mites were found in 15 samples from eight sites of four rivers (Mijares, Cabriel and Magro rivers and the tributaries of Júcar and Serpis rivers) and both water mites parasitizing pupae of Simuliidae and free-living water mites were found in four samples from four sites of three rivers (Cenia and Mijares rivers, and the Cabriel and Magro rivers, tributaries of Júcar river).

### Black fly—water mite relationship

**Fig. 2:**
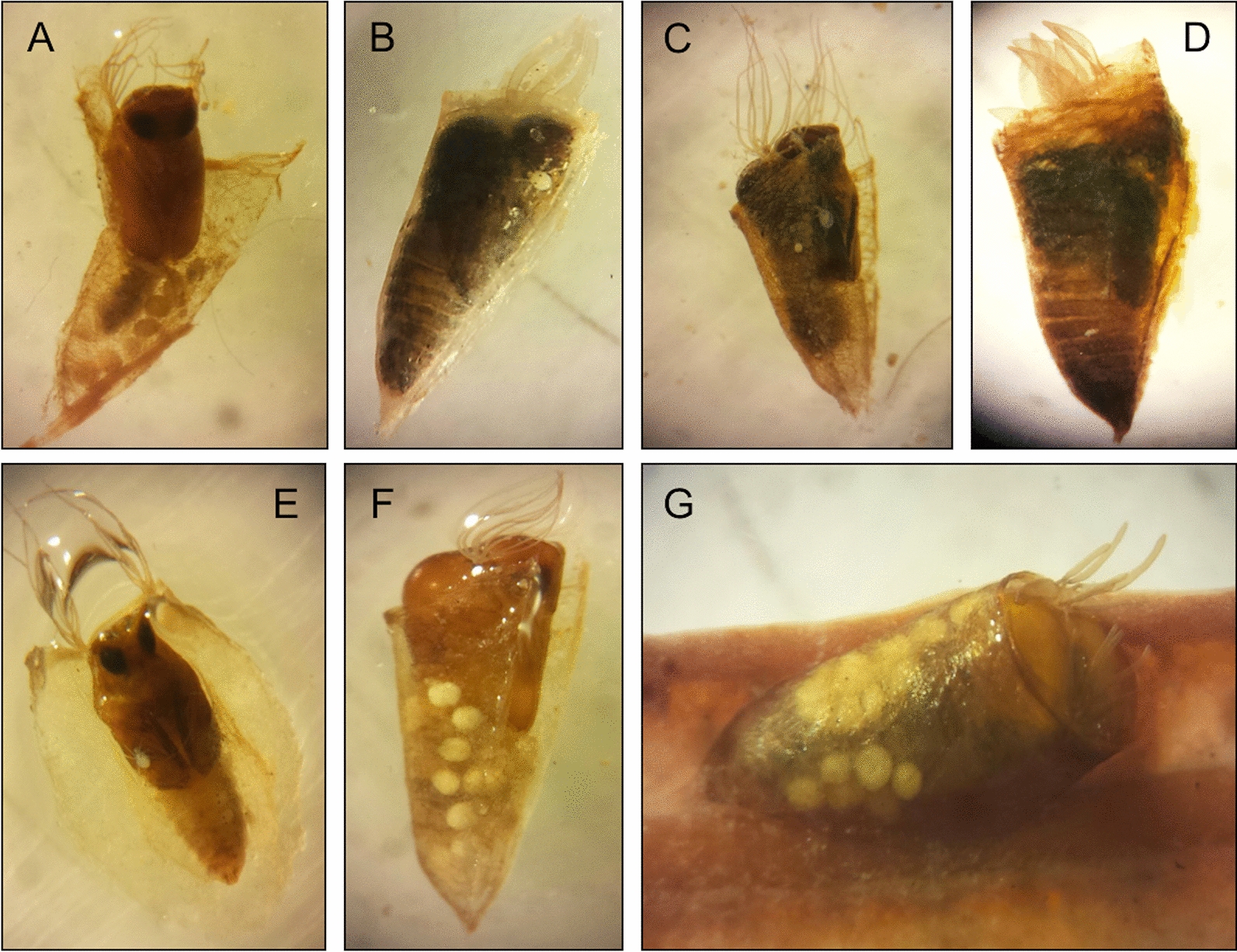
Species of black fly in pupa stage parasitized by water mites of the genus *Sperchon*.** a** *Simulium ornatum*,** b** *S. sergenti*,** c** *S. intermedium*,** d** *S. equinum*,** e** *S. angustitarse*,** f** *S. trifasciatum*,** g** *S. pseudequinum*. Photographs taken by D. López-Peña

**Fig. 3:**
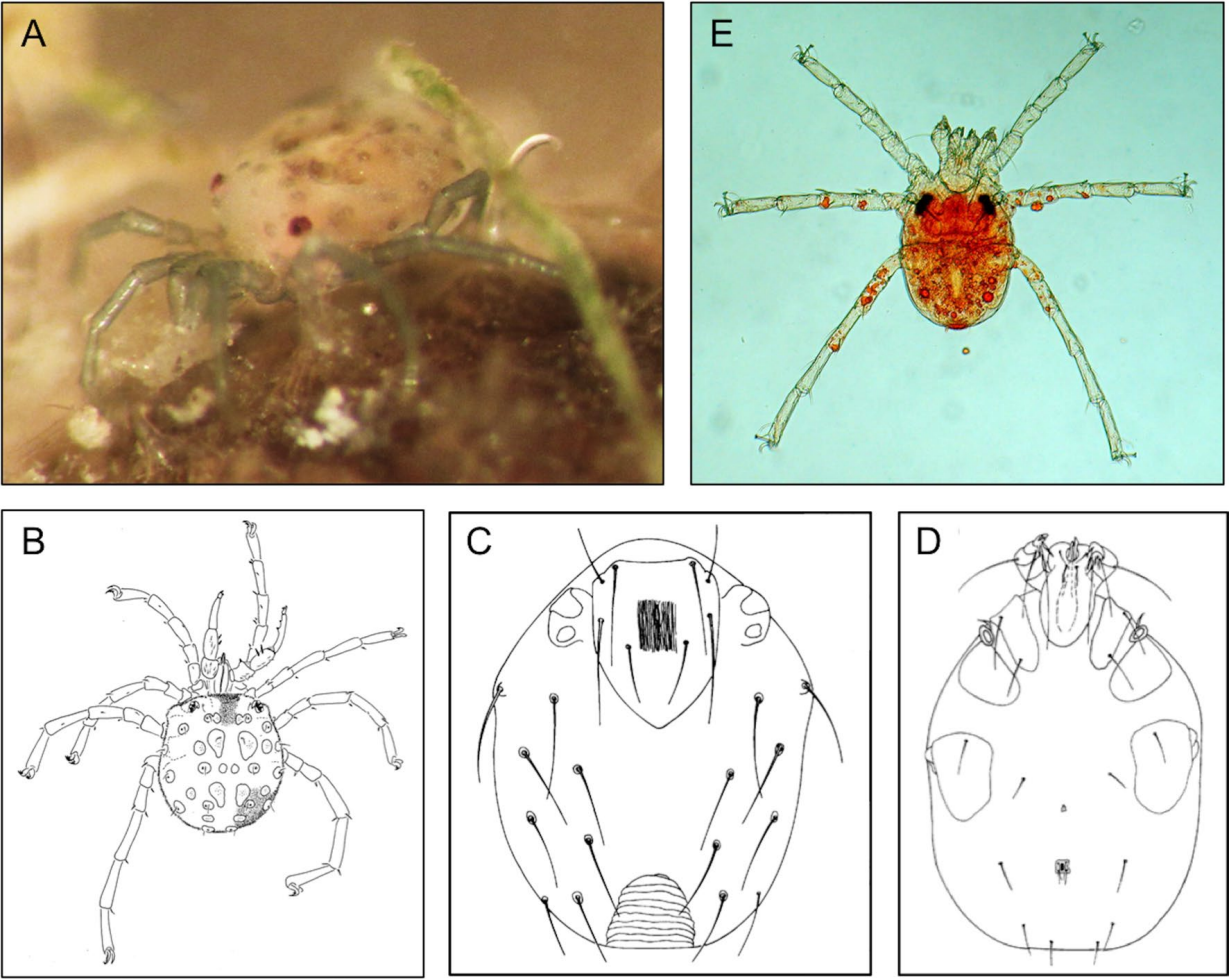
**a, b** Adults of *Sperchon setiger*, specimens from Germany: **a** fronto-lateral, **b** dorsal (note the setal pair on palp segment 3, typically present in *S. setiger* as well as in *S. algeriensis*). **c**–**e** Larvae of *Sperchon setiger*: **c** dorsal idiosoma, **d** ventral idiosoma and gnathosoma (**c**, **d** from [51]), **e** total, dorsally. Scale bars: 100 µm. **a**, **b** provided by R Gerecke, **c**, **d** provided by P Martin, **e** provided by A Renz

Of a total of 15,396 black fly pupae isolated from the samples, 426 were parasitized (2.8% of total black fly pupae isolated). Water mite infection was not equally distributed among black fly species (Table 2): only eight of the 21 black fly species identified were parasitized by water mites, including *Simulium* (*Nevermannia*) *angustitarse* (Lundström, 1911), *S.* (*Wilhelmia*) *equinum* (Linnaeus, 1758), *S.* (*Simulium*) *intermedium* Roubaud, 1906, *S.* (*Wilhelmia*) *lineatum* (Meigen, 1804), *S.* (*Simulium*) *ornatum* Meigen, 1818, *S.* (*Wilhelmia*) *pseudequinum* Séguy, 1921, *S.* (*Wilhelmia*) *sergenti* and *S.* (*Simulium*) *trifasciatum* Curtis, 1839. Water mites were found mainly in the space between the pupa and the pupal case, although some were also recorded on the pupal cases (Fig. 2). A relatively low percentage (< 30%) of these larvae was freshly hatched and unengorged, often co-existing in the same pupal cocoon with slightly to distinctly engorged specimens. In no case was attachment of larvae to the pupal skin observed. All mite larvae extracted from simuliid cocoons were found to be morphologically homogeneous, in agreement with the description given for *S. setiger* by Ullrich [26] and Martin [51] (lacking seta C3 on coxal plate II; small dimension of the dorsal plate; Fig. 3). However, in view of the above-described unclear taxonomic situation, water mite larvae will be addressed here generally as “*Sperchon* sp.” as further research is needed in order to clarify if these larvae do in fact represent *S. setiger* (a species in this study found in a few specimens only), or another *Sperchon* species (eventually *S. algeriensis*, the dominant *Sperchon* in this study and still unknown at the larval stage). Since the very most larval specimens were supposed to be in a pre-parasitic stage awaiting hatching of the adult black fly hosts, in the following text the mite larvae are termed as parasites—regardless of whether they may have engorged while attached to black fly pupae.

**Table 2:** Prevalence data on black fly species parasitization by water mites in eastern Spain

Black fly species	Number of parasitized pupae	Total number of pupae	Number of samples with parasitized pupae	Total number of samples
*Metacnephia blanci*	0	150	0	4
*Simulium angustipes*	0	19	0	10
*S. argygreatum*	0	1	0	1
*S. bertrandi*	0	2	0	3
*S. bezzii*	0	2	0	2
*S. carthusiense*	0	1	0	11
*S. cryophilum*	0	35	0	8
*S. erytrocephalum*	0	27	0	4
*S. petricolum*	0	284	0	22
*S. quadrifilia*	0	6	0	4
*S. reptans*	0	397	0	17
*S. velutinum*	0	65	0	13
*S. xanthinum*	0	8	0	7
*S. angustitarse*	1	1163	1	55
*S. sergenti*	1	1284	1	43
*S. lineatum*	7	997	4	14
*S. ornatum*	15	796	4	70
*S. intermedium*	35	3451	5	74
*S. pseudequinum*	292	5800	13	55
*S. equinum*	29	315	2	16
*S. trifasciatum*	46	582	6	33

**Fig. 4:**
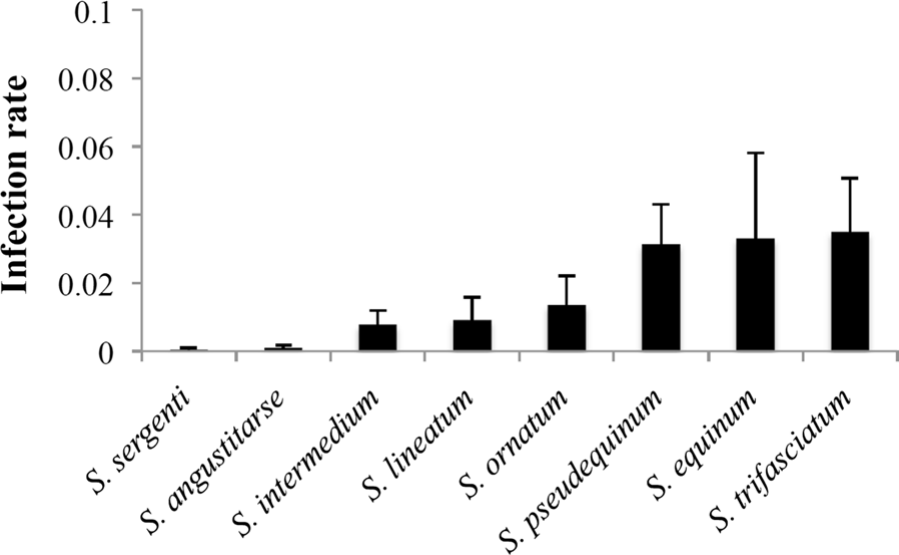
Infection rate (i.e. fraction of host population parasitized averaged per sample) of black fly species in which water mites were found. Error bars represent standard errors

Prevalence of parasitism differed among black fly species (Binomial GLM: deviance = 411.03,* df* = 20, *P*-value < 0.001) and was generally low (Fig. 4), with the highest values (± SD) being around 3% in *S. trifasciatum* (3.5 ± 1.6%), *S. equinum* (3.3 ± 2.5%) and *S. pseudequinum* (3.1 ± 1.2%). Even for these three* Simulium* species, the infection rate was unevenly distributed, and in many samples no parasites were found at all. The prevalence of water mite parasitism differed significantly among river basins in all the eight species parasitized (*P*-value < 0.001 for the “basin” factor in all cases after binomial GLM analyses). Table 3 shows the prevalence data according to river basins, with the Cenia and Mijares rivers being the most affected by: (i) the total number of pupae of black fly species parasitized; (ii) the incidence of sampling sites where parasites were detected; and (iii) the number of black fly species parasitized. Regarding the geographical distribution of parasitism by* Simulium* species, *S. intermedium* was found parasitized in three rivers (Cenia, Mijares and Algar rivers), while *S. equinum*, *S. lineatum* and *S. sergenti* were each found in only one river (Mijares, Júcar and Serpis rivers, respectively). The other four affected* Simulium* species were found in only two rivers each: *S. angustitarse* was found parasitized in the Cenia and Serpis rivers, *S. ornatum* and *S. trifasciatum* in the Cenia and Mijares rivers and *S. pseudequinum* in the Mijares and Júcar rivers.

**Table 3:** Prevalence data of black fly species parasitization by water mites by river basin

Basin^a^	Number of parasitized pupae	Total number of pupae	Number of samples with parasitized pupae	Total number of samples	Black fly species (*Simulium* spp.) parasitized	Total number of black fly (*Simulium*)species
Algar	1	369	1	5	*S. intermedium*	8
Amadorio	0	9	1	0	-	2
Cenia	6	659	2	2	*S. intermedium* *S. ornatum* *S. trifasciatum*	5
Cérvol	0	1	0	1	–	1
Júcar	22	2717	4	20	*S. lineatum* *S. pseudequinum*	16
Mijares	395	4575	11	17	*S. equinum* *S. intermedium* *S. ornatum* *S. pseudequinum* *S. trifasciatum*	13
Montnegre	0	208	0	4	–	7
Palancia	0	914	0	13	–	12
Serpis	2	3660	2	37	*S. angustitarse* *S. sergenti*	14
Turia	0	2284	0	16	–	11

^a^Basins are presented in alphabetical order

**Fig. 5:**
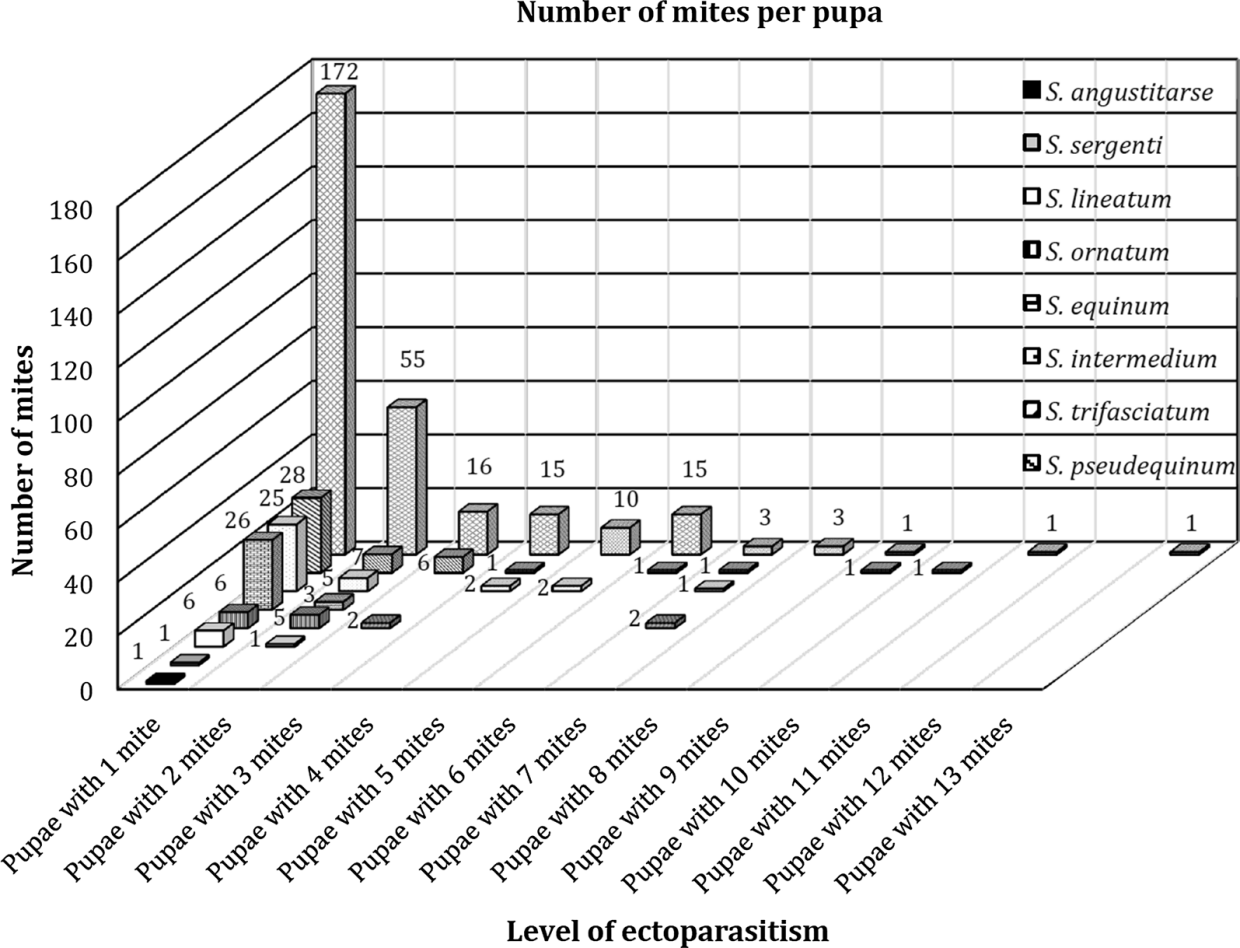
Number of parasitized pupae of black fly species and intensity of water mite parasitism

In terms of parasitism intensity, the load of water mites per pupae ranged from 1 to 13. However, this range was not homogeneous, and there was both intra- and interspecific variation in the number of water mite parasites present in black fly cocoons (Fig. 5). Significant differences were observed among species (Poisson GLM: deviance = 32.95,* df* = 7, *P*-value = 0.001). *Simulium pseudequinum* was the most affected of the eight black fly species. No significant correlation was found between the prevalence and intensity of parasitism for any of the infected black fly species, nor was there any relationship between these two variables and the abundance of black fly pupae at the sampling points.

Multiple logistic regression analysis revealed subsets of environmental variables explaining the prevalence of water mite parasitism for the different black fly species found (Table 4). The analyses were reduced to testing the effects of elevation, pH, conductivity, turbidity and oxygen concentration, since other variables measured in the field were highly correlated (*R*^2^ > 0.8) with other variables of this subset. The prevalence of water mite parasitism was significantly affected by any of these environmental variables only in four of the parasitized black fly species. Absence of significant relationships occurred in *S. angustitarse* and *S. sergenti* (both with very low values of parasite prevalence) and in *S. equinum*, for which the environmental dataset was limited (some variables could not be measured in the field due to logistic reasons and the number of sampling points where the species was present was scarce). Parasitism prevalence in *S. lineatum*, *S. ornatum*, *S. intermedium* and *S. trifasciatum* was significantly affected by pH. Notably, the coefficient estimates of the variable pH were negative in all cases, indicating that, in general, an increase in pH will be associated with a decreased probability of black fly species being parasitized by *Sperchon* sp. water mites. Further, for *S. lineatum*, *S. pseudequinum* and *S. trifasciatum*, pH was again the most significant environmental variable explaining water mite load per individual (Table 5). The sign of other environmental variables with significant effects on prevalence or intensity of parasitism was variable depending on each species, and no general pattern could be derived.

**Table 4:** Results of multiple logistic regression of environmental variables on prevalence of water mite parasitism on the pupae of black fly species

Species	Elevation	pH	Conductivity	Turbidity	Oxygen concentration
*S. angustitarse*	− 0.06	− 754.76	− 44.42	− 2.66	− 3.73
*S. sergenti*	0.01	− 9.43	6.14	− 0.16	− 1.16
*S. lineatum*	− 3.05	− 3953.30*	1650.98	262.20	–
*S. ornatum*	0.24	− 3529.00*	− 156.50*	− 0.06	35.82
*S. intermedium*	− 0.01	− 1583.00*	447.00*	− 19.88*	− 12.83
*S. pseudequinum*	− 0.01	6.72	7.79*	0.04	− 3.15
*S. equinum*	− 0.01	− 26.02	–	–	–
*S. trifasciatum*	0.60	− 4639.54*	− 397.89*	− 2.99*	− 23.10

Data are the beta regression coefficients

*Statistically significant after Bonferroni correction (α’ = 0.00625)

**Table 5:** Results of multiple logistic regression of environmental variables on intensity of water mite parasitism on the pupae of black fly species

Species	Elevation	pH	Conductivity	Turbidity	Oxygen concentration
*S. lineatum*	0.01	− 73.11	–	–	–
*S. ornatum*	0.01	0.22	–	–	–
*S. intermedium*	0.01	4.51	− 0.16	–	–
*S. pseudequinum*	− 0.03	− 54.31*	10.99	2.55	− 0.33
*S. trifasciatum*	0.02	− 32.77*	− 0.24	–	–

Data are the beta regression coefficients

*Statistically significant after Bonferroni correction (α’ = 0.00625)

### Free-living water mite assemblage study

Free-living deutonymphs and adults of water mites were recovered from only 15 of the 94 sampling sites (Table 6) and included a total of 78 specimens of true water mites, representing 15 different species of six genera and five families; two unidentified specimens of Oribatida were also collected (Table 7). The order Trombidiformes was represented by five families (indet. specimens are counted as separate species when obviously not belonging to one of the listed taxa): Hygrobatidae with four species (*Atractides nodipalpis* Thor, 1899, *Hygrobates calliger* Piersig, 1896, *H. fluviatilis* (Ström, 1768), and *H.* sp.); Aturidae with two species (*Aturus gallicus* Viets, 1939 and *A.* sp.); Lebertiidae with two species (*Lebertia porosa* Thor, 1900 and *L.* sp.); Sperchontidae with four species (all of the subgenus *Hispidosperchon*: *Sperchon algeriensis* Lundblad, 1942, *S. compactilis* Koenike, 1911), *S. denticulatus* gr. and *S. setiger* Thor, 1898) and Torrenticolidae with two species (*Torrenticola barsica* (Szalay, 1933) and *Torrenticola* sp.). In this study, the abundance of aquatic mites is referred to the immediate space where their (potential) hosts are present, above all the pupa stage, and the space between their body and the cocoon, but not to the whole river section in question. As a consequence, the representativeness of water mite data presented here is biased by the collection methodology used. A greater diversity of water mite species as well as a precise relative abundance would be expected applying a specific methodology for the collection of mites.

**Table 6:** Abundance of free-living mites in the study sites, mainly at the adult or deutonymphal stage

Sample number	Mite species	Male (*n*)	Female (*n*)	Deutonymph (*n*)	Larvae (*n*)	Mite total number
3	*Atractides nodipalpis*	1				1
	*Sperchon compactilis*	2				2
	*Sperchon setiger*			2		2
5	*Sperchon denticulatus* gr			1		1
	*Sperchon compactilis*	1		1		2
7 (3)	*Sperchon compactilis*		2	1		3
	*Torrenticola barsica*		1			1
14 (2)	*Sperchon compactilis*		2	1		3
	*Sperchon algeriensis*			1		1
15	Oribatida		1			1
17	*Hygrobates calliger*	1	2	2		5
	*Hygrobates* sp.			4		4
	*Sperchon algeriensis*	1				1
	*Sperchon denticulatus* gr			1		1
18 (2)	*Sperchon compactilis*	2	2	1		5
19	*Hygrobates fluviatilis*	2				2
	*Hygrobates* sp.			5		5
	*Aturus* sp.		1			1
	*Sperchon algeriensis*	2	2	13		17
20	*Torrenticola* sp.			1		1
	*Lebertia porosa*		1			1
	*Sperchon algeriensis*			2		2
	*Hygrobates* sp.			1		1
21	*Hygrobates* sp.			1		1
	*Sperchon setiger*			1		1
21 (2)	*Sperchon algeriensis*	1	2	4		7
	*Torrenticola barsica*	1				1
	*Hygrobates* sp.			1		1
22	*Aturus gallicus*	1	1			2
	*Sperchon algeriensis*		1			1
22(2)	Oribatida			1		1
24	*Lebertia* sp.				1	1
24 (2)	*Sperchon algeriensis*			1		1

**Table 7:** Counts of free-living water mite species ordered by taxonomic order and family

Order	Family	Species	Abundance	*n*^a^
Oribatida	–	–	2	2
Trombidiformes	Aturidae	*Aturus* sp.	1	1
		*Aturus gallicus* Viets, 1939	2	1
	Hygrobatidae	*Atractides nodipalpis* Thor, 1899	1	1
		*Hygrobates* sp.	12	5
		*Hygrobates calliger* Piersig, 1896	5	1
		*Hygrobates fluviatilis* (Ström, 1768)	2	1
	Lebertidae	*Lebertia* sp.	1	1
		*Lebertia porosa* Thor, 1900	1	1
	Sperchontidae	*Sperchon algeriensis* Lundblad, 1942	30	7
		*Sperchon compactilis* Koenike, 1911	15	5
		*Sperchon denticulatus* gr	2	2
		*Sperchon setiger* Thor, 1898	3	2
	Torrenticolidae	*Torrenticola* sp.	1	1
		*Torrenticola barsica* (Szalay, 1933)	2	2

^a^*n* denotes the number of sampling points where each species was found, and information regarding their location and physico-chemical variables can be found in Table 1

For most of these species, data on larval morphology and host preference are completely lacking (*Aturus gallicus*, *Sperchon algeriensis, S. compactilis*, *Torrenticola barsica*), or the data are not trustworthy due to unclear taxonomic state (*Atractides nodipalpis*, *Hygrobates calliger* and *Lebertia porosa* are representatives of groups of sibling species in the course of revision (V Pešić and R Gerecke, unpublished). As the taxonomic attribution of populations from the Iberian Peninsula is unclear in many cases, the host range also needs to be reconsidered at a lower geographic scale.

Free-living stages of water mite species were not homogeneously distributed. The vast majority of the identified water mite species were singletons (i.e. species reported just from one sample), as was the case for *Atractides nodipalpis*, *Aturus* sp., *A. gallicus*, *Hygrobates calliger*, *H. fluviatilis* gr., *Lebertia* sp., *L. porosa* and *Torrenticola* sp. The most diverse and abundant share of free-living individuals belonged to the family Sperchontidae, with *S. algeriensis* as the most abundant species (30 specimens) and present in the highest number of samples (7), followed by *S. compactilis* (15 specimens from 5 different samples). Postlarval specimens of *S. setiger*, the only species previously reported parasitizing black fly pupae, were also present in some samples, although it was neither remarkably abundant nor common in the assemblages of free-living adult water mites.

### Sequencing and genetic distance

The most frequent *Sperchon* species in the study area, *S. algeriensis*, was originally described from northern Africa (*Sperchon* (*Hispidosperchon*) *algeriensis* Lundblad, 1942) and subsequently recorded from many sites in the central and western Mediterranean Palaearctic area [34]. Here we present for the first time molecular data for a population of this species, collected in the proximity of Villalonga, a town located on the banks of the Serpis river. This analysis was made possible due to the “DNA-Eco” project coordinated by Vladimir Pešić (University of Montenegro, Podgorica), and sequencing was done at the Canadian Centre for DNA Barcoding (CCBD, Guelph, ONT, Canada; http://ccdb.ca/; sample ID: CCDB 41824 E04; BOLD ID: HYDBH052-22). The results of this analysis confirm that *S. algeriensis* is a distinct species, clearly separable from *S. compactilis* and *S. setiger* (with the latter probably being an aggregate of 2 or more species). Furthermore, the high genetic distance of 15.4% between the Spanish material and a specimen from Iran, attributed to *S. algeriensis* by Pešić et al. [52], suggests that the latter belongs to a further distinct species. It likely represents *S. beneckei* Bader & Sepasgosarian, 1982, a species proposed to be a synonym of *S. algeriensis* by Asadi et al. [53].

## Discussion

The ecological importance of water mites in freshwater lotic ecosystems has often been inappropriately underestimated in ecological studies [21]. Far beyond their diversity and abundance, water mites can exert important effects on lotic community structure as predators, parasites or both [25, 54]. Black fly species may constitute a biomedical and veterinary problem of growing concern [33] as potential hosts. They can be parasitized mostly as imagos following the hatch, to which water lice larvae are attached following parasitism during the pupal stage of the black fly [19]. Therefore, in this study we provide information on the prevalence and intensity of water mite parasitism on black fly pupae in the field for a broad span of territory in eastern Spain where the abundance of black fly species is already beginning to be treated as a public health issue [33, 55–58].

Overall, we report a relatively low prevalence of water mite parasitism on black fly pupae, with the highest values being approximately 3%, which agrees with other observations, such as in chironomids [59–61]. Most of the species of black flies were not parasitized at all, and in the parasitized species, water mite larvae were found only on a low proportion of pupae. Notwithstanding, several aspects are remarkable. First, some species of black flies showed a significantly higher level of parasitism than others, as also reported previously by other authors (see [62]). In our study, this was the case for *Simulium trifasciatum*, *S. equinum* and *S. pseudequinum*, the latter two species characterized by their hematophagic habits and veterinary importance. Showing a lower prevalence of parasitism were two other species of biosanitary concern, namely *S. ornatum* (approx. 2%) and *S. lineatum* (< 1%). Second, a single water mite species of the genus *Sperchon* was probably responsible for all the black flies specimens parasitized in our study. Larvae of *Sperchon* species typically suck hemolymph from their adult hosts [19, 62, 63]. Interestingly, mite larvae in the black fly pupal cocoons were found in states ranging from the unfed to engorged, suggesting that they used black fly pupae to obtain food. Regarding the specificity of the host-parasite relationship, our results are qualitatively consistent with previous findings by Gledhill et al. [19], who also reported cases of *S. equinum* and *S. ornatum* parasitized by water mites attributed to *S. setiger* in southern England, although with a higher prevalence in *S. ornatum*. Regarding water mite load per pupae, the highest number of *Sperchon* larvae found in the present study was described for a *S. pseudequinum* pupa with 13 mites, while Gledhill et al. [19] reported an approximately twofold higher maximum number of mites in *S. ornatum*. For adult simuliids, Ullrich [27] reported an average of about four larvae attached to simuliid imago, reaching a maximum of 21 larvae per host. Intensity rates were highest in emergence peaks of the preferred host species. Since a high intensity of parasitism probably deeply affects the vitality of the host individuals, food intake in the pupal simuliids (visible in the engorged water mite larvae) might be an alternative way of survival for the water mite larvae in periods when too many mite larvae and too few simuliids are present in the field. Insect larvae and/or pupae are generally seldom used as water mite hosts. For example, for *Arrenurus* (*Megaluracarus*) *globator* (Müller, 1776), a species with an unusually broad host spectrum, both larvae and adults of nematocerans were found as host [64]. Larvae of the water mite genus *Unionicola*, which are typical parasites of adult chironomids, were found additionally found attached to caddisfly larvae [65].

Several factors may affect the prevalence and intensity of parasitism of water mites on black fly pupae. On one hand, it has been suggested that the morphology of the pupae is a determining factor for some black fly species to be potential hosts of *S. setiger* [19]. Such specificity may be related to the thickness of the pupal respiratory filaments. In this context, Gledhill et al. [19] suggested that thinner respiratory filaments facilitate the infestation by water mite larvae. This notion holds true in our study for black fly species with thin respiratory filaments, such as *S. ornatum*, *S. intermedium*, *S. pseudequinum* and *S. trifasciatum* (also heavily affected in the study by Gledhill et al. [19]), but not for *S. equinum* (whose respiratory filaments are quite thick and short). Davies [62] suggested that the prevalence of parasitism may be related to both factors, namely the ease of access into the pupal chamber and the amount of stored nutrients in fat bodies or maturing eggs. This observation infers the possibility that *Sperchon* water mites tend to pre-select their hosts on the basis of some kind of chemical attraction, a matter for further research ([19], but see also [27]).

On the other hand, prevalence and intensity of parasitism by water mite larvae may be differentially affected by ecological factors. Most water mites are known to be very vulnerable to modifications in substrata, water quality or discharge [66] and also to be affected by the different ecological demands of their developmental stages. Therefore, a detailed study of the relative abundance of the specimens of each species collected in the different substrates of the river frequented, together with the ecological factors of the habitats, such as the physical–chemical nature of the water, could shed more light on these mites. However, according to Renz et al. [20], levels of infestation seem to be linked to water pollution, and these authors reported that infestation levels were lowest in rivers with a low organic load. In Spain, we observed that parasitism prevalence in *S. lineatum*, *S. ornatum*, *S. intermedium* and *S. trifasciatum* was negatively affected by pH, and this same environmental variable explained water mite load per individual in *S. lineatum*, *S. pseudequinum* and *S. trifasciatum*. Roughly, our results suggest a trend to lower prevalence of water mite parasitism with increasing values of pH, conductivity and turbidity, factors which ultimately impair water quality. Specifically free-living stages of water mite species were also negatively correlated with pH. Further, our results suggest a positive relationship between black fly pupae abundance and prevalence. Thus, we conjecture that the higher prevalence of parasitism by water mites is due to a density-dependent effect, by which greater overcrowding would favor transmission, but it is generally not well understood how water mite larvae search for their potential hosts in lotic environments [12].

Natural species assemblages of free-living stages of water mites are the potential sources from where black fly parasites could emerge. Typically, different assemblages are associated to varying ecological features of the stream habitats and ultimately depend on the ecological preferences of each species. Water mites are known to be among the most diverse of freshwater organisms [67], with more than 370 species described in Spain (Iberian Peninsula, Balearic Islands and Canary Islands) [68] and, for example, more than 450 species described in Central Europe [69]. However, species richness is generally reduced in running waters with strong seasonal changes [70]. The species that parasitized black fly pupae in our study is a representative of the genus *Sperchon*. *Sperchon setiger*, the species that so far has attracted the most interest as a parasite of black flies, is present in the study area, but the species most frequently encountered in the assemblages of free-living stages of water mites is *S. algeriensis*. The latter is reported from Sicily as a character species of summer-warm Mediterranean streams with temporaneous surface flow [70], but its larval morphology and life-cycle are still unknown. In fact, the hydrography of the present study area is characterized by summer drought. Therefore, the high abundance of this species in the present study is not surprising because of the presence of intermittent habitats. Along with some other species of the subgenus *Hispidosperchon*, adults and deutonymphs of *S. algeriensis*, *S. setiger* and *S. compactilis*, also species recorded in our study, share a series of morphological similarities. Further research on the life-cycles and larval morphology is required in order to better understand the parasite-host relationship of all these species. Questions that naturally arise concern the general host preference of the involved species. Also, we cannot exclude that we have to deal with a set of cryptic species differing in host preference. To the contrary, we may be dealing with a single species that parasitizes a much wider range of dipteran taxa and is able to switch, following ecological conditions, from one host to another. It is likely that in the natural habitats where free living stages of water mites are found, a wider choice of flying-host items may be available in varying proportions for these parasites; for example, several species in the genus *Sperchon* are known to use chironomids as their hosts as well [16, 17], which Martin [18] showed for *S. setiger*.

## Conclusions

The main aim of this study was to get insight into the parasitic relationship of water mites (Hydrachnidia) with black flies. The results show that these Hydrachnidia affect the pupal stage of different species of simuliids. A variation in the load of water mites per pupae was detected at both intra- and interspecific level. The data reported here contribute to a better understanding of the water mite–black fly relationship, the prevalence of parasitism, bioecology and geographical distribution of water mites.

## Data Availability

All data analyzed during this study are included in this published article. Simuliids and most water mite voucher specimens are stored in the Colección de Entomología de la Universitat de València (Estudi General); selected water mite specimens are stored in the collection Reinhard Gerecke (Tübingen), all in properly labeled vials.

## Abbreviations

BOLD System: Barcode of Life Data System
CCA: Canonical correspondence analysis
COI: Cytochrome oxidase I gene
DCCA: Detrended canonical correspondence analysis
GLMs: Generalized linear models
RDA: Redundancy analysis
